# The Effects of Probiotic/Synbiotic on Serum Level of Zonulin as a Biomarker of Intestinal Permeability: A Systematic Review and Meta-Analysis

**DOI:** 10.18502/ijph.v49i7.3575

**Published:** 2020-07

**Authors:** Amirhossein RAMEZANI AHMADI, Mehdi SADEGHIAN, Meysam ALIPOUR, Samira AHMADI TAHERI, Sepideh RAHMANI, Amir ABBASNEZHAD

**Affiliations:** 1. Department of Nutrition, School of Allied Medical Sciences, Ahvaz Jundishapur University of Medical Sciences, Ahvaz, Iran; 2. Clinical Research Development Unit, Golestan Hospital, Ahvaz Jundishapur University of Medical Sciences, Ahvaz, Iran; 3. Food Security Research Center, Isfahan University of Medical Sciences, Isfahan, Iran; 4. Nutritional Health Research Center, Lorestan University of Medical Sciences, Khorramabad, Iran

**Keywords:** Probiotic, Synbiotic, Gut barrier, Intestinal permeability, Zonulin

## Abstract

**Background:**

This systematic review and meta-analysis was conducted to obtain a conclusive result on the influence of probiotics/synbiotic on serum levels of zonulin. Data related to serum levels of zonulin were extracted to determine the effects of probiotic/synbiotic on intestinal permeability.

**Methods:**

The literature search was conducted across the Cochrane Central Register of Controlled Trials, Pub-Med, Scopus and ISI Web of Science, Search up to Nov 2018. Clinical trials evaluating the effect of probiotic/synbiotic on serum zonulin levels of all human subjects were included.

**Results:**

Nine studies (including 496 intervention and 443 control subjects) met the inclusion criteria for the meta-analysis. According to the meta-analysis, probiotic/synbiotic has a significant effect on serum zonulin reduction (WMD=−10.55 [95% CI: −17.76, −3.34]; *P*=0.004). However, the high level of heterogeneity was observed among the studies (I^2^=97.8, *P*<0.001). The subgroup analysis suggested study quality, blinding, study duration, Participants age, subject’s health status and supplement type as sources of heterogeneity.

**Conclusion:**

Probiotic/synbiotic have favorable effects on serum levels of zonulin as a measure of intestinal permeability. However, the results should be interpreted with caution due to the high heterogeneity and further evidence is required before definitive recommendations can be made.

## Introduction

The gastrointestinal epithelium, covered by a single layer of epithelial cells, forms a protective barrier between apical and basolateral compartments. The function of the epithelial barrier mostly relies on intercellular junctions, known as tight junctions which consist of cytoplasmic plaque proteins including zonula occludens (1). One of the modulators of these tight junctions is Zonula occludens toxin (Zot) which temporarily increases the paracellular permeability without damage to the epithelium (2). Zonulin, a 47-kDa protein, is an endogenous analog of Zot that can similarly improve paracellular transport to Zot (3). Zonulin represents a physiological defensive mechanism against microorganism colonization of the small intestine (4). Altered intestinal permeability in several pathological conditions including autoimmune diseases, diseases of the nervous systems, and neoplastic conditions has been associated with overexpression of zonulin in the intestinal mucosa (5). Moreover, antigen presentation in human macrophages appears to be regulated by zonulin. This would change the cytokine profile and subsequently potentiate the switch from immune tolerance to autoimmunity (6).

Strategies aimed at modifying the intestinal barrier function through downregulating zonulin pathway suggest a potential therapeutic target for the treatment of these chronic diseases. Zonulin inhibitor Larazotide acetate featured an upcoming result in celiac disease; however, safety and efficacy of Larazotide need to be determined by large clinical trials (7). Besides conventional treatments, several nutritional compounds including Colostrum bovinum (8), Apple-Derived Pectin (9), vitamins A and D (10) have been found to modulate the epithelial barrier by reducing serum levels of zonulin. Probiotic (live microorganisms) and synbiotic (containing probiotic strains and prebiotics including inulin, starch, and fructooligosaccharides) are promising groups of nutraceuticals that affect intestinal permeability through alterations in expression of tight junction proteins (11). Several studies have been conducted to investigate the impacts of probiotics on serum or fecal levels of zonulin; however, the results were inconsistent and inconclusive (11, 12). Therefore, we conducted this systematic review and meta-analysis to review systematically the influence of probiotics or synbiotic on serum levels of zonulin.

## Materials and Methods

This research conducted by following the guidelines and the PRISMA statement for reporting systematic reviews and meta-analysis. Table 1 outlined the PICOS (population, intervention, comparator, outcome, and setting) criteria used to perform the systematic review. Due to the study type, ethical approval was not necessary according to local legislation.

**Table 1:** PICOS (population, intervention, comparator, outcome, and setting) criteria used to perform the systematic review

***PICOS***	***Criteria***
Population	All human subjects
Intervention	Probiotic/synbiotic supplementation
Comparator	Control group (placebo or without intervention)
Outcome	Serum level of zonulin
Setting	Randomized Clinical Trials

### Search strategy

Two researchers (MA and AR) independently searched databases including the Cochrane Central Register of Controlled Trials, PubMed, Scopus, ISI Web of Science, and google scholar for clinical trials that investigated the effect of probiotic/synbiotic on serum levels of zonulin. The search included all studies published as original full-text articles covering a period up to Nov 2018. The literature search was conducted using the following keywords and medical subject heading (MeSH) terms in any possible combination: probiotic*, symbiotic, synbiotic, prebiotic, “lactic acid bacteria”, Streptococc*, “S. thermophiles”, Bifidobacter*, Lactobacill*, Lactococc*, Saccharomyces, Bacillus, “B. mesentericus”, Enterococc*, “E. faecium”, “B. clausii”, Clostridium, “C. butyricum”, “Escherichia coli Nissle”, “E. coli Nissle”, VSL#3, bifidu*, pediococc*, LGG, Rhamnosos, Reuteri, Acidophilus, Lactis, Plantarrum, Bulgaricus, Johnsonii, Ecologic, Faecalis, zonulin, “intestinal permeability”, “GI permeability”, “gastrointestinal permeability”. No restriction was applied to publication year, and all studies published in English were included. The reference lists of included studies were investigated to identify any additional relevant studies. The title and abstract of the search output were screened by two reviewers separately and potentially relevant studies were identified.

### Study Selection

After that the relevance of a study was confirmed, publication’s full texts were reviewed and those that fulfilled the eligibility criteria were included. The following eligibility criteria were applied: 1) published in English or Persian; 2) using probiotic/synbiotic as the supplement; 3) reporting serum zonulin as the outcome of the study. Following studies were excluded: 1) articles without full-text availability, non-English, ecological study, qualitative study, opinion pieces, conference abstracts, review articles and editorials; 2) reporting unrelated data.

### Data Extraction

The data were extracted independently by 2 reviewers (MA and AR), and in the event of disagreement, a decision being made after MS cross-examined doubtful data. Studies characteristics including first author’s name, publication year, country, study design, quality score, sample size, supplement and placebo composition, study duration, participant’s gender, age, and health status were extracted. Moreover, to evaluate the effect of probiotic/synbiotic on zonulin concentration, the mean ± SD of the serum zonulin before and after supplementation was extracted from eligible studies.

### Assessment of risk of bias

The quality of the studies was evaluated by 2 separate reviewers according to the Jadad score (13). The Jadad score considers randomization, blinding, description of withdrawals and dropouts. Each study was scored between 0–5; higher numbers represent better quality.

### Statistical analysis

The effect size, estimated as the weighted mean difference (WMD), was used to perform the fixed method meta-analysis. In case of significant heterogeneity between studies, a random-effects meta-analysis was carried out (14). Heterogeneity was evaluated using the I^2^ index and Cochrane’s Q test. Heterogeneity was considered low if I^2^ < 30%, moderate if I^2^ = 30%–75%, and high if I^2^ >75% (15). Subgroup analyses were performed according to study quality (low or high), blinding (yes or no), age (under 45 yr or more than 45 yr), supplement type (probiotic or synbiotic), study duration (less than 3 months or more than 3 months), and participant’s health status (healthy or not healthy) to identify the potential sources of heterogeneity. In addition, sensitivity analysis and meta-regression were performed to investigate further on heterogeneity sources. Begg’s rank correlation, Egger’s linear regression, and funnel plots were used to examine for the presence of publication bias. All analyses were carried out using Stata, ver. 14 SE (Stata Crop, College Station, TX, USA). *P-*values <0.05 were considered statistically significant.

## Results

### Characteristics of the studies

**Fig. 1:**
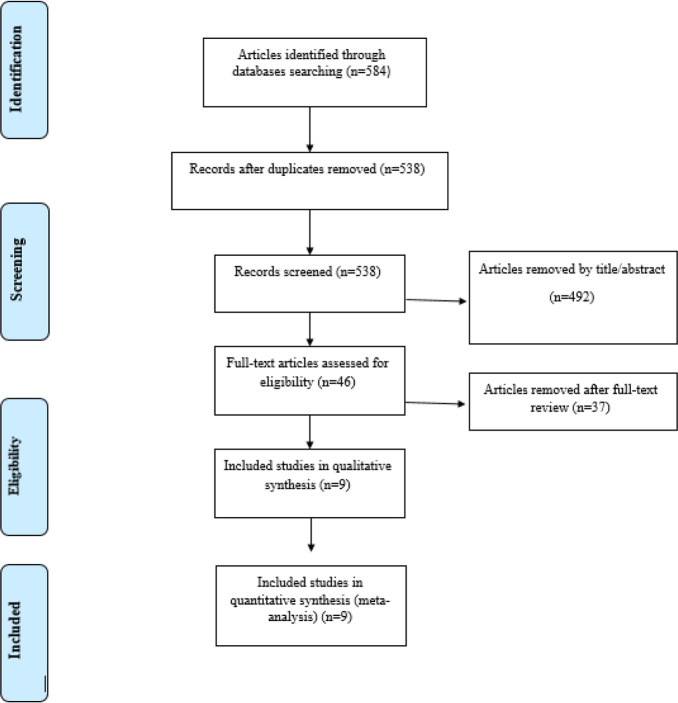
Flow chart of the process of the study selection

As shown in Fig. 1, the early electronic search resulted in 538 studies, after duplicate removal. Following the title and abstract screen, 492 studies were excluded due to reporting unrelated data, being review articles, and not be written in English.

Overall, 46 studies were evaluated for eligibility, and 37 studies were excluded for the following reasons: did not report serum levels of zonulin as study outcome, did not provide enough data, or was published as study protocol. Nine studies met the inclusion criteria for the meta-analysis (16–24). Table 2 summarizes the characteristics of all studies included in the systematic review. All trials had a parallel study design. Studies were conducted in China (23, 24), Finland (17, 22), Netherlands (20, 21), Turkey (18) and the USA (16). Moreover, one of the studies was multicentric with different countries (19). Most of the studies recruited both male and female gender, while two studies were conducted only in males (16) or females (17). The duration of the intervention ranged from 14 to 180 days. Four studies recruited healthy subjects (16, 17, 19, 21).

**Table 2:** Characteristics of included studies

***First author, publication year***	***Country***	***Sample size (M/F)***	***Mean age (yr)***	***RCT design (blinding)***	***Duration (days)***	***Supplement content***	***Comparison group***	***Health status***	***Jaded score***
Liu, 2013 (23)	China	78/72	62.2	Parallel (Yes)	16	Lactobacillus plantarum, Lactobacillus acidophilus-11, Bifidobacterium longum-88	Maltodextrin	Colorectal carcinoma	4
Liu, 2015 (24)	China	70/64	60.1	Parallel (Yes)	16	LP, LA-11, BL-88	Maltodextrin	Colorectal liver metastases	4
Stenman, 2016 (22)	Finland	31/103	50.0	Parallel (Yes)	180	Group1: LU Group 2: B420 Group3: LU+B420	Microcrystalline cellulose	Overweight and Obese	5
Wilms, 2016 (21)	Netherlands	11/9	20.7	Parallel (Yes)	14	Synbiotic	Maltodextrin	Healthy	2
Çakır, 2017 (18)	Turkey	18/10	12.2	Parallel (No)	120	Synbiotic	No placebo	NAFLD	1
de Roos, 2017 (20)	Netherlands	56/4	39.9	Parallel (Yes)	84	Probiotic mixture	Maize starch, Maltodextrin	Migraine	4
Kantah, 2017 (19)	Multicenter	N/A	50	Parallel (No)	150	Group 1: Synbiotic Group 2: Probiotic Group 3: Synbiotic + Probiotic	Marine PUFA extract	Healthy	3
Mokkala, 2018 (17)	Finland	-/101	30.4	Parallel (Yes)	147	Bifidobacterium animalis ssp. Latics 420 and Lactobacillus rhamnosus HN001	Microcrystalline cellulose	Pregnant	5
Townsend, 2018 (16)	USA	25/-	20.1	Parallel (Yes)	84	Bacillus subtilis DE111	Maltodextrin	Baseball Players	4

RCT, randomized controlled trial; M, male; F, female; NR, not reported; NAFLD, non-alcoholic fatty liver disease;

Other studies were conducted on colorectal carcinoma (23), Colorectal liver metastases (24), overweight or obese (22), non-alcoholic fatty liver disease (18), and migraine patients (20). According to Jadad scores, 7 studies were classified as high-quality papers (score≥3) (16, 17, 19, 20, 22–24) and 2 studies were classified as low-quality (18, 21).

### Findings from the meta-analysis

**Fig. 2:**
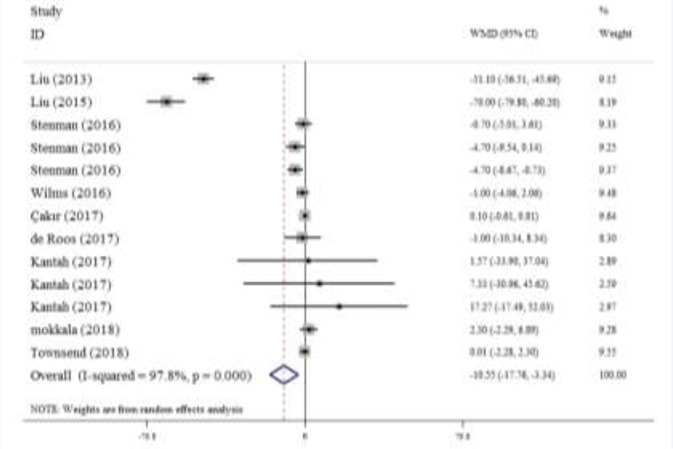
Forest plot of trials examining the effect of probiotic/symbiotic on serum zonulin

Overall, 9 studies provided 13 effect sizes, including 496 intervention and 443 control subjects, regarding the effect of probiotic/synbiotic supplementation on the serum levels of zonulin. According to the meta-analysis, probiotic/synbiotic has a significant effect on reducing serum zonulin compared to the placebo groups (WMD=−10.55 [95% CI: −17.76, −3.34]; *P*=0.004; Fig. 2). However, the high level of heterogeneity was observed among the studies (I^2^=97.8, *P*<0.001).

The subgroup analysis showed that heterogeneity was significant in high-quality studies (n=11, I^2^=97.9, *P*<0.001), with blinding (n=9, I^2^=98.4, *P*<0.001), less than 3 months of study duration (n=5, I^2^=99.1, *P*<0.001), subjects with age more than 45 yr (n=8, I^2^=98.2, *P*<0.001), studies that enrolled not healthy subjects (n=7, I^2^=98.9, *P*<0.001), and trials that supplemented by probiotic (n=9, I^2^=98.3, *P*<0.001) (Table 3).

**Table 3:** Overall estimates of meta-analysis on the effect of probiotic/synbiotic on the serum zonulin

***Outcomes***	***Subgroups***	***No. of effect size***	***References***	***WMD (95% CI)***	***P-value***	***I^2^ (%)***	***P-value for heterogeneity***	***Meta-regression***	
								***Coefficient (95% CI)***	***P-value***
Zonulin (ng/ml)		13	( 16– 24)	−10.55 (−17.76, – 3.34)	0.004	97.8	<0.001		
Study quality	High	11	( 16, 17, 19, 20, 22–24)	−11.87 (−23.88, 0.13)	0.053	97.9	<0.001	19.26 (−59.18, 97.72)	0.570
	Low	2	(18, 21)	0.04 (−0.64, 0.73)	0.900	0.0	0.493		
Blinding	Yes	9	(16, 17, 20–24)	−14.14 (−25.15, −3.14)	0.012	98.4	<0.001	−7.24 (−59.97, 45.49)	0.748
	No	4	(18, 19)	0.11 (−0.59, 0.81)	0.760	0.0	0.782		
Intervention duration	<3 mo	5	(16, 20, 21, 23, 24)	−24.37 (−45.36, −3.37)	0.023	99.1	<0.001	33.23 (0.30, 66.1)	0.048
	≥3 mo	8	(17–19, 22)	−0.09 (−0.76, 0.57)	0.785	37.4	0.131		
Age	< 45 yr	5	(16–18, 20, 21)	0.08 (−0.57, 0.73)	0.805	0.0	0.838	−27.08 (−67.41, 13.24)	0.151
	≥ 45 yr	8	(19, 22–24)	−16.15 (−34.75, 2.44)	0.089	98.2	<0.001		
Supplement Type	Probiotic	9	(16, 17, 20, 22–24)	−14.56 (−27.35, −1.78)	0.026	98.3	<0.001	18.26 (−56.42, 92.94)	0.572
	Synbiotic	4	(18, 19, 21)	0.05 (−0.63, 0.74)	0.883	0.0	0.701		
Health status	Healthy	6	(16, 17, 19, 21)	0.07 (−1.62, 1.77)	0.933	0.0	0.782	−9.58 (−45.89, 26.71)	0.542
	Not healthy	7	(18, 20, 22–24)	−18.51 (−32.34, −4.69)	0.009	98.9	<0.001		

Sensitivity analysis suggested that this association disappeared following the study of Liu et, al. (24) was omitted (WMD=−5.55 [95% CI: −11.76, 0.65]). According to meta-regression, there was significant evidence for an association between study duration and the effect of probiotic/synbiotic on serum levels of zonulin (Coefficient=33.23, 95% CI=0.30, 66.16, *P*=0.048). However, study quality, blinding, supplement type, participants’ age, and health status were not found to be associated with this relationship (Table 3).

**Fig. 3:**
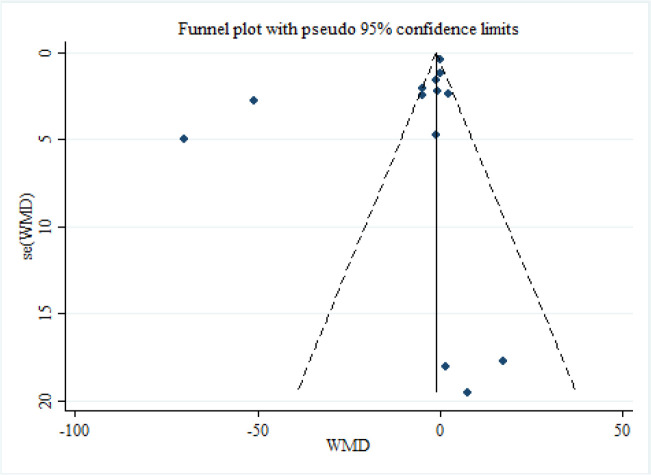
Funnel plots detailing publication bias in the studies selected for analysis. Visual inspection of funnel plots indicating that there is no publication bias among studies

Visual inspection of the funnel plot (Fig. 3) demonstrated no publication bias of trials that investigated the effect of probiotic/synbiotic supplementation on the serum levels of zonulin (Egger’s test P=0.154; Begg’s test *P*=0.502).

## Discussion

Findings of the current meta-analysis suggest that compared to placebo, supplementation with pro-biotic/synbiotic significantly reduces serum levels of zonulin; however, there was high heterogeneity among selected studies. When the analysis was separately performed for probiotic and synbiotic, a significant reduction was observed in those that received probiotic only. Moreover, subgroup analysis based on study quality, blinding, duration of intervention, age, and health status could explain the source of heterogeneity among studies. Zonulin is considered as a physiological modulator of intestinal permeability and a surrogate marker of the dysfunctional gut barrier (3, 5, 25). Certain gut microbes, in particular pathogens, might induce the release of zonulin from the gut suggesting a mechanistic link between alterations in the gut microbiota and gut barrier function (4). Despite the small sample size, our finding is of importance due to the increased levels of circulating zonulin in a wide range of chronic diseases (26–28). Although we observed heterogeneity among studies, the results were not significant when the analysis was confined to high-quality studies, blinded trials, studies with longer duration, and those recruited healthy subjects younger than 45 yr old. The supplemented probiotics may surpass certain lactic acid bacteria that activate Toll-like receptor 2 (TLR2) signaling pathway (12). TLR2 is localized in cell membranes of the intestinal epithelium. It induces epithelial resistance in the activated form (29, 30). Moreover, protective role of probiotics on intestinal permeability may be explained in part by inhibiting p38 MAPK, a Ser/Thr kinase related to the upregulation of several inflammatory indices (23). In vitro studies assumed that the combination of probiotics with prebiotics might change intestinal permeability simultaneously (31, 32). Therefore, we confined the analysis to supplemental synbiotic and observed that the results were no longer significant. Because of varying study populations and probiotic products, more powerful clinical trials with longer follow-up are needed to confirm this finding.

Sensitivity analysis indicated that results might have been disproportionally influenced by a study (24). After excluding this trial, the meta-analysis no longer produced significant results that might be responsible for the obtained results. This might be due to a considerable weight of mean difference derived from lower reported values for mean serum zonulin and small standard deviation. Moreover, the study was conducted on patients with colorectal liver metastasis (CLM) that have higher postoperative levels of serum zonulin rather than colorectal cancer patients without liver metastasis (23). Therefore, there may be a liver barrier playing a role in the change of postoperative zonulin levels in CLM. When we examined the association between study duration and the effect of probiotic/synbiotic on serum levels of zonulin, we found that studies with longer duration of intervention had lower serum levels of zonulin.

Some strengths should be highlighted in this meta-analysis. We used a rigorous search strategy and a systematic methodology based on the current guidelines for systematic reviews to determine the effects of supplemental probiotic/synbiotic on serum levels of zonulin, a hallmark of intestinal permeability associated with several chronic conditions. However, several limitations of this study are fully acknowledged. While studies indicated that different species of probiotics act differently in reserving tight junction integrity and barrier function (33), we could not perform subgroup analysis based on probiotic type due to limited number of studies. Furthermore, we could not analyze fecal concentrations of zonulin due to the lack of reported data in this regard. Since the gut is not the only source of circulating zonulin, data on the association of serum levels of zonulin and intestinal zonulin are ambiguous (3). Nevertheless, the decreased ratio of lactulose/mannitol, an indicator of intestinal permeability, was associated with reduced levels of serum zonulin (23).

## Conclusion

Oral probiotic supplementation has favorable effects on serum levels of zonulin, a measure of intestinal permeability, although the results should be interpreted with caution due to the influence of a single study on the pooled effect size. High heterogeneity was noted and further evidence is required before definitive recommendations can be made.

## Ethical considerations

Ethical issues (Including plagiarism, informed consent, misconduct, data fabrication and/or falsification, double publication and/or submission, redundancy, etc.) have been completely observed by the authors.
